# Secretory carcinoma of the breast with multiple distant metastases in the brain and unfavorable prognosis: a case report and literature review

**DOI:** 10.1186/s13000-021-01115-1

**Published:** 2021-06-24

**Authors:** Hongping Tang, Lihua Zhong, Hongbing Jiang, Yan Zhang, Guannan Liang, Guoyan Chen, Gui’e Xie

**Affiliations:** 1 Department of Pathology, Affiliated Shenzhen Maternity & Child Healthcare Hospital, Southern Medical University, 518028 Shenzhen, China; 2 Department of Breast Surgery, Affiliated Shenzhen Maternity & Child Healthcare Hospital, Southern Medical University, 518028 Shenzhen, China; 3 Department of Radiology, Affiliated Shenzhen Maternity & Child Healthcare Hospital, Southern Medical University, 518028 Shenzhen, China; 4 Department of Pathology, Shenzhen Longhua District Maternity & Child Healthcare Hospital, 518109 Shenzhen, China; 5 KingMed School of Laboratory Medicine, Guangzhou Medical University, 510182 Guangzhou, China

**Keywords:** breast cancer, secretory carcinoma, chemotherapy, case report

## Abstract

**Background:**

Secretory carcinoma of the breast is one of the rarest entities, accounting for less than 0.15 % of all infiltrating breast carcinomas. It has characteristic histopathological and molecular features and, in general, a more favorable prognosis. In this case report, we describe a local, advanced secretory carcinoma of the breast with aggressive course and an unfavorable outcome.

**Case presentation:**

A hard, painless, and palpably bossed mass approximately 12.0 cm in diameter occupied most of the left breast of a 39-year-old woman with fixation to the overlying skin. Breast ultrasonography and magnetic resonance imaging (MRI) scans gave the same grading as BI-RADS IV. A needle biopsy was performed, and the pathological diagnosis was secretory carcinoma. Neoadjuvant chemotherapy (NAC) was then performed, after which ultrasonography and MRI scans revealed chemo-resistance of the tumor to NAC. Left breast mastectomy and axillary lymphadenectomy were subsequently performed. Tumor cells were triple-negative and positive for S-100 and periodic acid-Schiff (PAS) staining. Fluorescence in-situ hybridization (FISH) analysis indicated a fusion arrangement of the ETV6-NTRK3 gene. The patient developed multiple distant metastases in the brain and died of these metastases 19 months after initial diagnosis.

**Conclusions:**

Secretory carcinomas of the breast have been described as a low-grade histologic subtype with a favorable prognosis. This case showed chemo-resistance to neoadjuvant chemotherapy, multiple distant metastases, and a final unfavorable outcome. Further research is needed to better understand the behavior and treatment of this rare tumor.

## Introduction

Secretory carcinoma is a very rare type of breast carcinoma. It was first reported in children and known as juvenile breast carcinoma [1], but now it is known to occur in adults of both sexes. Secretory carcinoma of the breast is known to demonstrate characteristic histopathological and immunohistochemical features [2]. Recently, it was shown that an ETS variant 6-neurotrophic tyrosine kinase receptor type 3 (ETV6-NTRK3) gene fusion was associated with secretory carcinoma [3, 4]. This carcinoma of the breast has generally been described as having a favorable prognosis, which has suggested that treatment should be as conservative and non-aggressive as possible [5].

Here, we report a case of a 39-year-old woman who suffered from local advanced secretory carcinoma of the breast with chemo-resistance to neoadjuvant chemotherapy, subsequent multiple brain metastases, and had an unfavorable outcome. The aim of this report is to help to better understand the behavior of this rare tumor and to develop a standard approach to treatment.

## Case presentation

### Clinical history

A 39-year-old woman noted a lump in her left breast for 13 months that had increased rapidly for 2 months. In May 2017, the patient was first admitted to our hospital. She had no past history of malignancy and no family history of breast carcinoma.

**Fig. 1:**
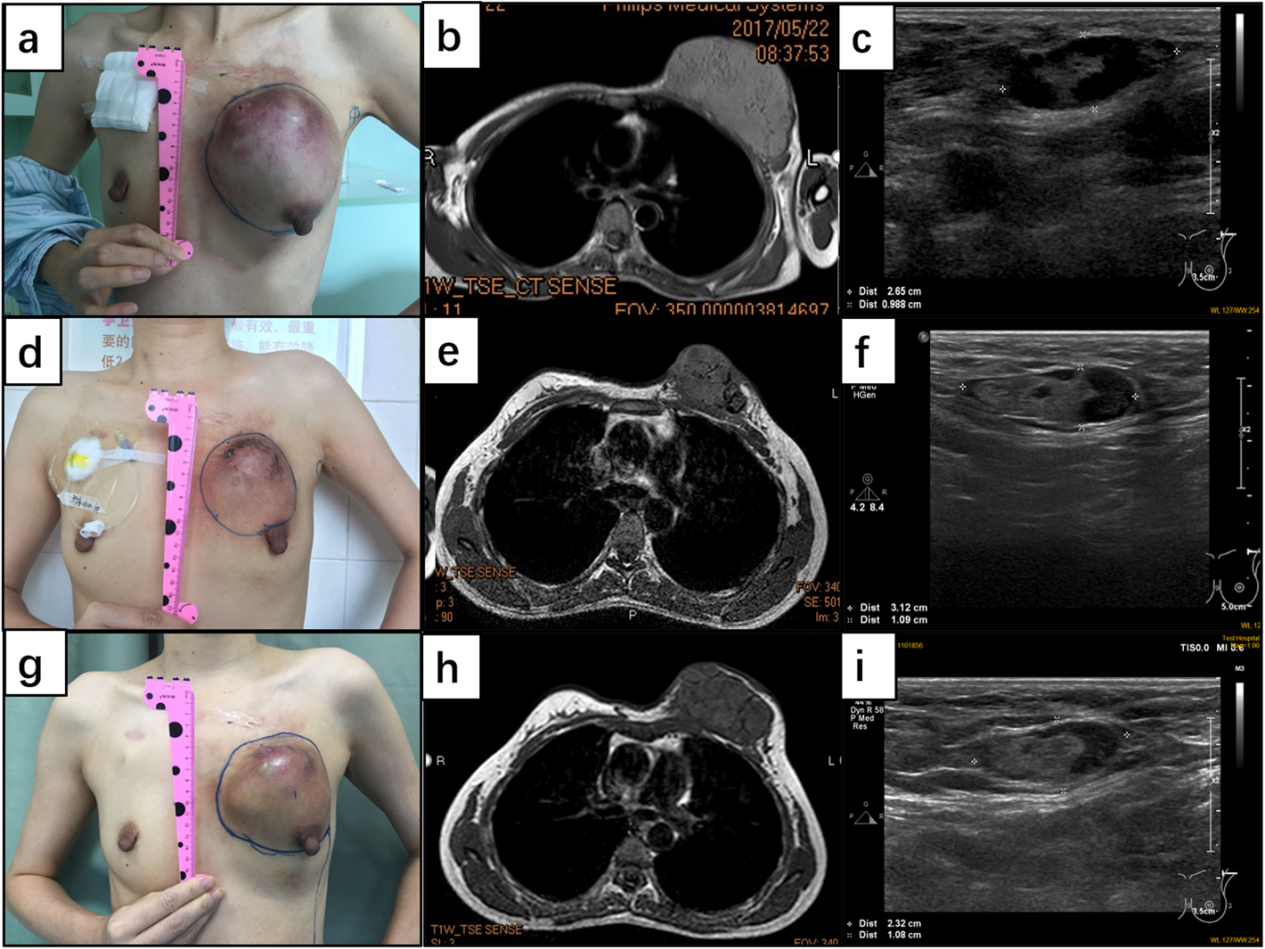
Physical, imaging and ultrasound examination of tumor during NAC. **a, b, c**: pre-chemotherapy; **d, e, f**: after 4th cycle of EC; **g, h, i**: 25 days after 2nd cycle of DC

On physical examination, a hard, painless and palpably bossed mass approximately 12.0 cm in diameter occupied most of the left breast with fixation to the overlying skin (Fig. 1a). No abnormalities were present in the right breast. An MRI scan revealed a focal, high-density mass of 12.0 × 11.0 cm, which was graded as BI-RADS IV (Fig. 1b). Breast ultrasonography revealed a sizeable mass of 11.0 × 11.0 cm, regularly shaped in the left breast and a hypoechoic mass approximately 2.8 × 1.0 cm in the left axilla. Ultrasonography examination also graded the breast mass as BI-RADS IV (Fig. 1c). The resulting pathological diagnosis following needle biopsy was secretory carcinoma. According to the AJCC Cancer Staging Manual [6], the disease in this patient was staged as T4N1M0 (Stage IIIB). Neoadjuvant chemotherapy was subsequently performed with 4 cycles of Epirubicin/Cyclophosphamide (EC) regimens and 2 cycles of Docetaxel/Carboplatin (DC) regimens. Routine physical examination was performed following each cycle of chemotherapy. As shown in Table 1, tumor size gradually decreased in the first 4 cycles of EC regimens. However, subsequently the tumor increased in size following 2 cycles of DC regimens. Figures of physical examination, MRI scan and ultrasonography during the whole course of neoadjuvant chemotherapy also revealed that the tumor was partially responsive to EC regimens therapy but progressed after DC regimens (Fig. 1a and i).

This patient completed modified radical mastectomy (left breast mastectomy and left axillary lymphadenectomy) and consecutive latissimus dorsi breast reconstruction in October 2017. Four cycles of Fluorouracil/Epirubicin/Cyclophosphamide (FEC) regimens were continuously administered. Radiotherapy was carried out on the chest wall and drainage areas (50 Gy/25 f). The patient was switched to intensive chemotherapy with oral Capecitabine for 8 cycles.

**Fig. 2:**
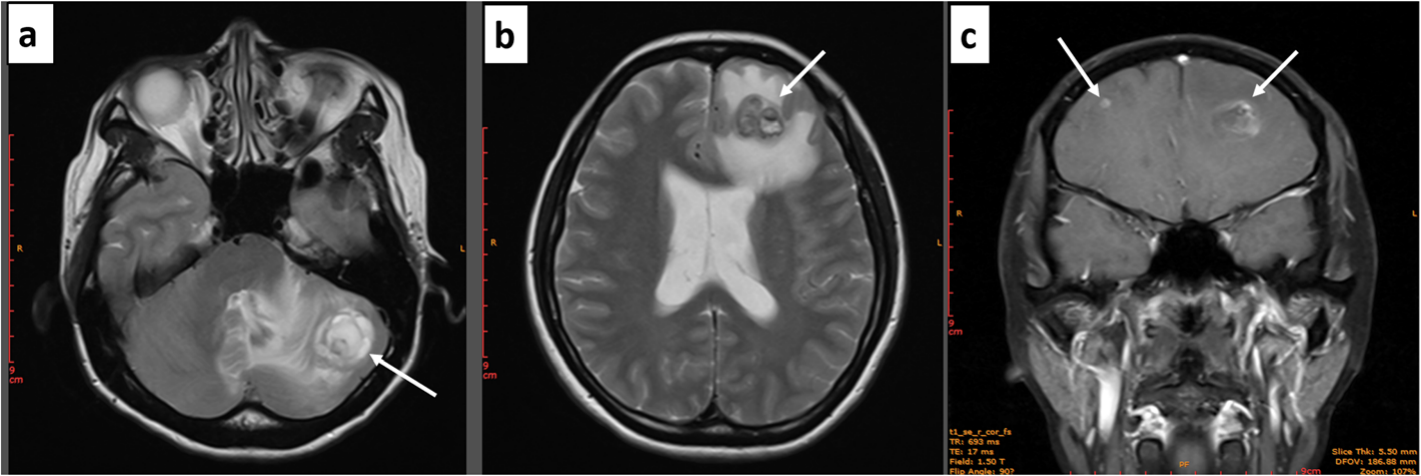
Multiple metastases (white arrows) in the brain as scanned by MRI. **(a)** metastases in the left cerebellar hemisphere in the horizontal plane; **(b)** metastases in the left frontal lobe in the horizontal plane; **(c)** metastases in the bilateral frontal lobe in the coronal plane

In October 2018, the patient was hospitalized again with complaints of headache and ataxia. A brain MRI scan showed multiple metastases in the left occipital lobe, bilateral frontal lobe, and left side of the cerebellum, with the largest mass having a maximum diameter of 3.5 cm (Fig. 2a-c). Whether there were metastases in other organs was unclear. The patient ended treatment and died in March 2019. The disease-free survival and overall survival for this patient were 10 months and 19 months, respectively.

**Table 1:** Tumor size by physical examination during NAC

Chemotherapy course	①	②	③	④	⑤	⑥	⑦	⑧
**Length (cm)**	14.0	13.0	12.0	10.0	10.0	9.0	10.0	11.0
**Width (cm)**	13.0	11.0	10.5	9.5	9.5	9.0	10.0	11.0
**Height (cm)**	8.0	6.0	4.5	4.0	3.5	4.0	4.5	5.0

① Pre-chemotherapy, ② after 1st cycle of EC, ③ after 2nd cycle of EC, ④ after 3rd cycle of EC, ⑤ after 4th cycle of EC, ⑥ after 1rst cycle of DC, ⑦ after 2nd cycle of DC, ⑧ 25 days after 2nd cycle of DC

### Gross features

**Fig. 3:**
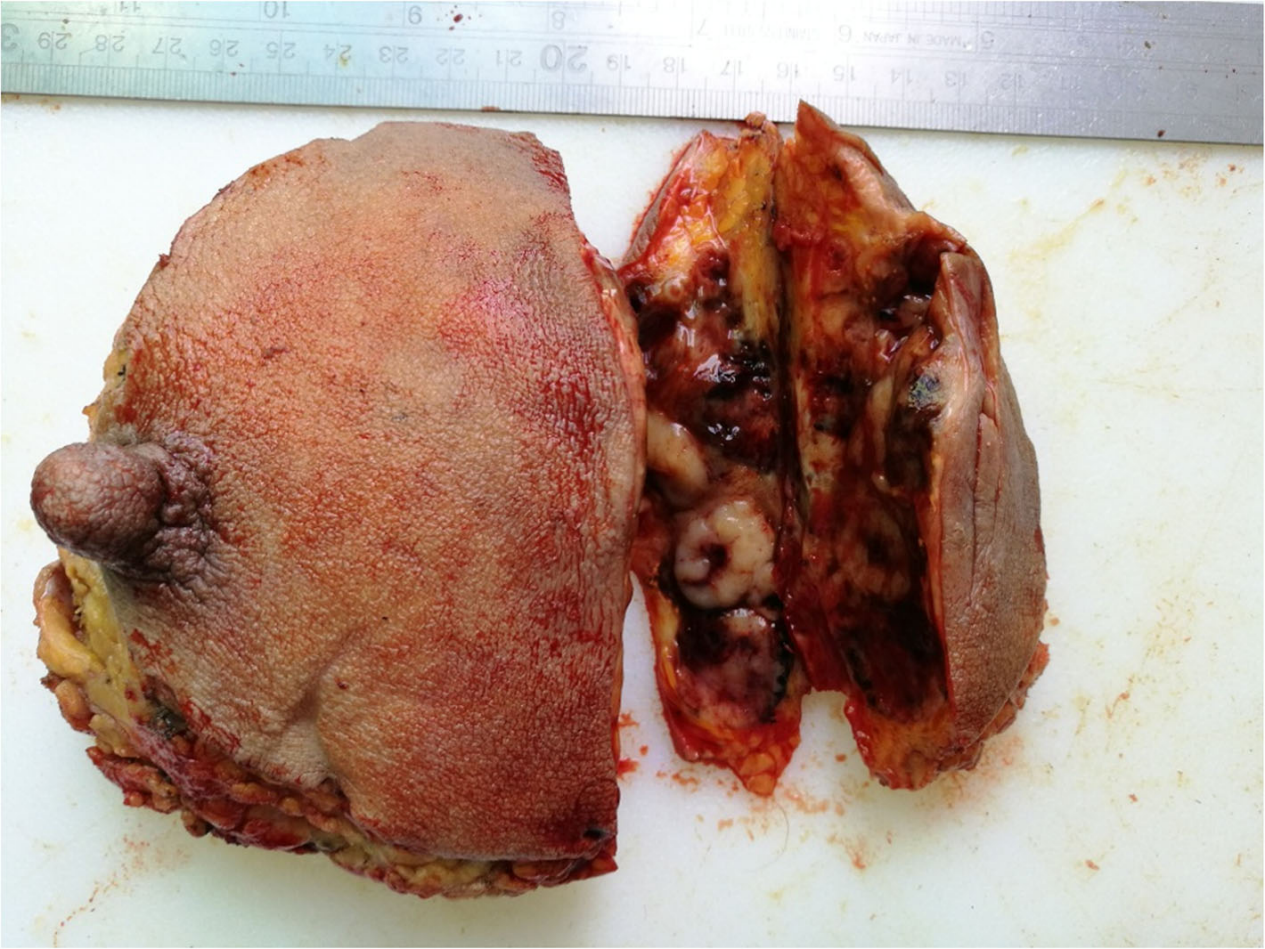
Gross finding appearance of the mastectomy specimen showed a solid-cystic mass with clear margins and white nodules on the cut surface

On gross examination, the needle biopsy specimen pre-chemotherapy was strip-like, gray-red, slightly hard in texture, with no special features otherwise apparent. The mastectomy specimen following chemotherapy was covered with skin, and the nipple was submitted for examination. The cut surface showed a mass of approximately 8 × 7 × 5 cm, with white-gray multiple nodules accompanied by prominent bleeding and necrosis. No nipple or periareolar lesions were seen (Fig. 3). A total of 15 lymph nodes were detected in the separate axillary tissues.

#### Microscopic features

**Fig. 4:**
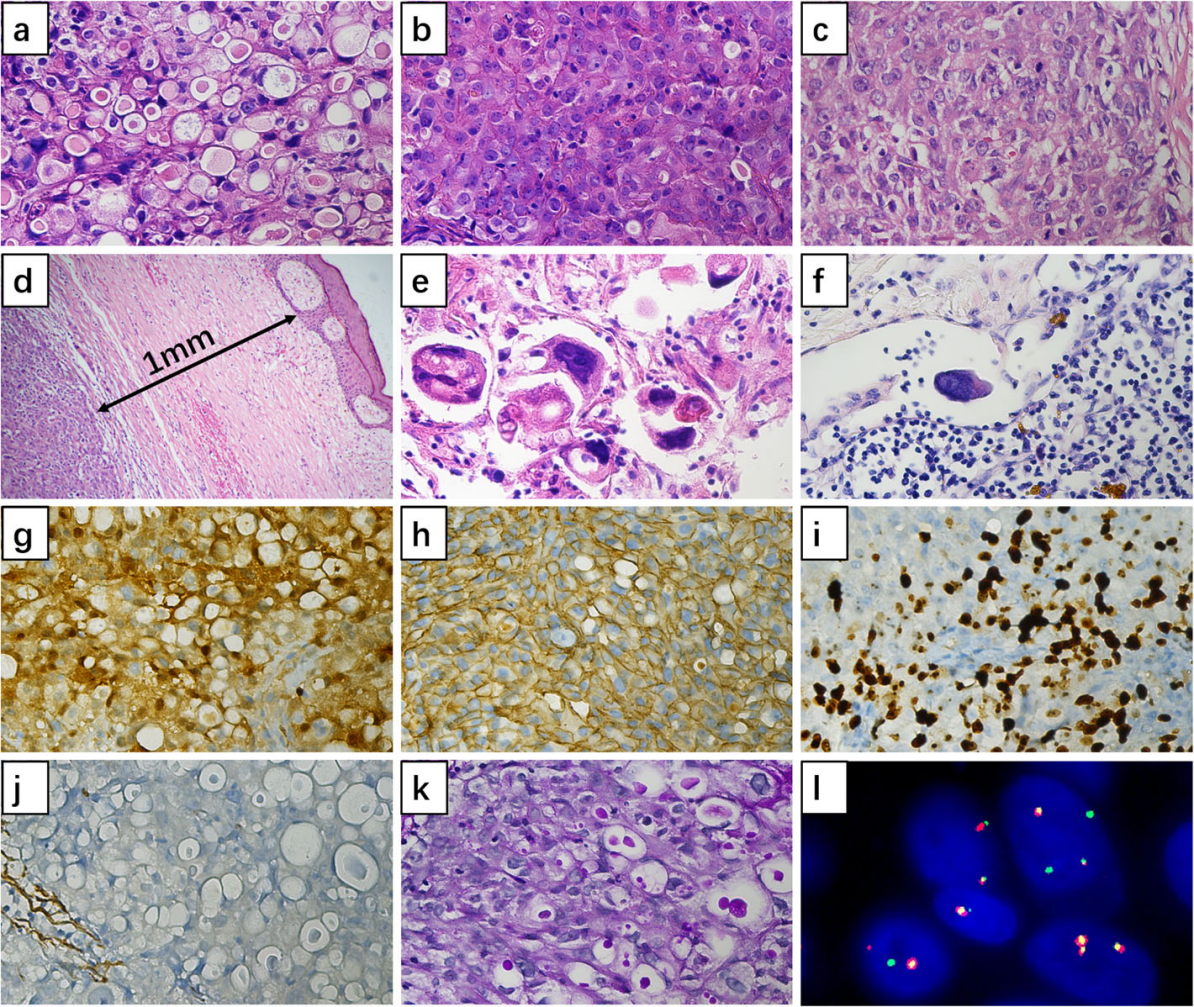
Microscopic features. **a**. Microcystic pattern of the tumor before chemotherapy (HE, ×400); b. solid pattern of the tumor before chemotherapy (HE, ×400); **c**. solid pattern of the tumor in the mastectomy specimen (HE, ×400); **d**. the pushing border near the skin shown in the mastectomy specimen (HE, ×100); **e**. morphological changes of tumor cells after chemotherapy (HE, ×400); **f.** isolated tumor cell was detected in axillary lymph node (HE, ×400); **g**. Positive cytoplasm and nuclear staining for S-100 by IHC (Envison, ×400) ; **h**. Positive cytomembrane staining for E-Cadherin by IHC (Envison, ×100); **i**. Positive nuclear staining for Ki-67 by IHC (Envison, ×400); **j**. Negative staining for SMA by IHC (Envison, ×400); **k**. Positive PAS staining of the extracellular secretory material (×400); **l**. ETV6-NTRK3 fusion arrangement indicated by FISH analysis

Microscopic examination of the biopsy specimen pre-chemotherapy revealed tumor cells arranged in combinations of microcystic (Fig. 4a) and solid patterns (Fig. 4b), which characteristically contained abundant extracellular secretory material. The cells had abundant granular eosinophilic cytoplasm with round moderate grade nuclei. There was no perineural or vascular invasion. The result of this biopsy therefore suggested secretory carcinoma of the breast. Examination of the mastectomy specimen showed that the tumor also displayed a combination of microcystic and solid patterns (Fig. 4c) and a pushing border near to the skin (Fig. 4d), accompanied by massive necrosis and hemorrhage. Responding to neoadjuvant chemotherapy, the tumor cells showed obvious morphological changes in certain areas, such as nuclear concentration and fragmentation of tumor cells, in addition to pleomorphic giant tumor cells with eosinophilic and foamy cytoplasm (Fig. 4e). An isolated tumor cell was detected in one enlarged axillary lymph node (Fig. 4f).

Immunohistochemistry of the biopsy specimen showed that the tumor cells had positive cytoplasmic staining for epithelial membrane antigen (EMA), S-100 (Fig. 4 g), vimentin, and positive cytomembrane staining for E-Cadherin (Fig. 4 h) while it was triple-negative for estrogen receptor (ER), progesterone receptor (PR) and human epidermal growth factor receptor type 2 (HER2). The specimen was also negative for androgen receptor (AR), gross cystic disease fluid protein 15 (GCDFP-15), and smooth muscle actin (SMA, Fig. 4j). The Ki-67 proliferation index of tumor cells was 40 % (Fig. 4 h). The secretory material was found to be positive for periodic acid-Schiff (PAS) staining (Fig. 4i).

Fluorescence in-situ hybridization (FISH) analysis indicated the fusion translocation t(12;15) of the ETV6-NTRK3 gene. As shown in Fig. 4 L, the red signal represents the ETV6 gene, while the green signal represents the NTRK3 gene. A number of ETV6-NTRK3 fusion signals were detected by FISH analysis.

## Discussion

Secretory carcinoma of the breast is one of the rarest subtypes, accounting for less than 0.15 % of all infiltrating breast carcinomas [2]. It was first described in 1966 by McDivitt and Stewart as a rare breast neoplasia identified in female children and adolescents, with an average age of nine [1]. However, it is now clear that secretory carcinoma can occur in adults of both sexes [7–9]. The typical clinical presentation of secretory carcinoma of the breast is a slow-growing, painless, well-circumscribed, palpable mass that can occur anywhere but is more common in the outer upper quadrant of the breast [10]. Microscopically, secretory carcinoma of the breast has been shown to be arranged with a solid, microcystic and tubular structure composed of cells that produce abundant intracellular and extracellular milk-like secretory material intensively positive for alcian blue or PAS. Tumor cells have low-grade features with small to medium size, oval to round nuclei, scant mitotic activity, and abundant granular eosinophilic cytoplasm. Occasionally, a papillary pattern can be the dominant architecture. Immunohistochemically, tumor cells are positive for S100 staining and triple-negative for ER, PR and HER2 [2, 11, 12]. In addition to the breast, secretory cancer can occur in other organs that contain secretory glands, such as parotid gland, salivary gland, sweat gland, lacrimal gland and thyroid [13–18]. Secretory carcinoma that occurs in other organs shows the same combined structures of cribriform patterns, solid patterns, microcystic patterns, and the extracellular secretion which is positive for Alcian blue or PAS.

Tognon et al. were the first to report in 2002 that secretory carcinoma of the breast harbored a recurrent balanced chromosomal translocation, t(12;15) (p13; q25), which leads to the formation of ETV6-NTRK3 fusion [12]. In addition to secretory carcinomas occurring in the breast, salivary gland [14] and thyroid [18], ETV6-NTRK3 gene fusion is also encountered in several neoplasms, including ALK-negative inflammatory myofibroblastic tumors, the cellular variant of congenital mesoblastic nephroma, congenital fibrosarcoma and radiation-induced papillary thyroid carcinoma [19, 20]. Meanwhile, some novel gene mutations of secretory carcinomas occurring in the salivary glands have been recently reported. Sasaki et al. [21] found a case with a CTNNA1-ALK fusion, and Black et al. [22] reported another case harboring two gene fusions, ETV6-RET and EGFR-SEPT14, expanding the molecular characterization of secretory carcinoma beyond the ETV6-NTRK3 gene mutation. A large-scale parallel sequencing analysis of 9 cases of secretory breast cancer, including one ductal carcinoma in situ, showed that there were no additional typical mutations in breast cancer except ETV6-NTRK3 gene mutation in all cases, while the mutation burden was very low [23].

Secretory carcinoma of the breast has generally been described as one with a favorable prognosis. However, several cases have presented distant metastases. To date, a total of 14 cases of secretory carcinoma of the breast with distant metastasis have been reported in the literatures (10 females and 4 males; 2 cases with disseminated metastases, 6 cases with multiple organ metastases), with ages ranging from 8 to 73 years (mean 36 years). The most common metastatic organs were the lung (9 cases), liver (4 cases), and bone (4 cases), with the other metastatic sites being the skin (2 cases), kidney (1 case), mediastinum (1 case), pancreas (1 case) and pleural (1 case). A total of 8 cases were reported to become fatal due to the metastatic secretory carcinoma. Mortality ranged from 6 to 240 months after initial diagnosis (mean survival time was 74.6 months) [5, 24–33]. As summarized by Hoda et al., there were no obvious clinicopathological features of secretory carcinomas, such as the patient’s gender, tumor location, imaging findings, follow-up treatment of surgery and/or chemotherapy, axillary node status, or the expression status of ER, PR and HER2, to indicate the distant invasion ability of the tumor [24]. Tavassoli and Norris [7] suggested that three features may indicate a favorable prognosis for patients with secretory carcinoma of the breast: (1) tumor size less than 2 cm; (2) age of less than 20 years at diagnosis; and (3) circumscribed margins. Unfortunately, none of these three features were present in our case. Moreover, relatively high Ki-67 proliferation index (40 %) and nuclear grade (intermediate) of tumor cells have been suggested to be associated with the poor prognosis of this patient, in consideration of the correlations between these two indicators and the prognosis of other breast cancers [34, 35]. Sequencing analysis has revealed only ETV6-NTRK3 mutation, with no additional molecular alterations in any of the reported cases of secretory carcinoma of the breast, whether with or without distant metastasis [24, 28, 30, 31]. Detection of additional gene mutations, such as TERT promoter mutation and CDKN2A/b deletion, may be helpful as an indicator for prediction of the invasive course of secretory breast cancer.

Due to the paucity of reported cases for secretory carcinoma, no consensus guidelines for treatment are available. However, surgery is considered the mainstay of treatment for secretory carcinoma. The demonstration of late local recurrence has led many to propose mastectomy for patients with this disease [36]. In adults, a simple mastectomy, at minimum, is recommended. Modified radical mastectomy has been favored by some authors in cases with tumor sizes greater than 2 cm and poor gross circumscription [37]. Adjuvant chemotherapy and radiation have been used as treatments; however, these therapies have had little to no success [28]. As a novel treatment strategy, targeted therapy of patients with NTRK fusion-positive cancers with a TRK inhibitor, such as larotrectinib or entrectinib, has been shown to be associated with high response rates [38]. Shukla et al. reported a successful targeted therapy experience using larotrectinib to treat refractory ETV6-NTRK3 fusion-positive secretory breast carcinoma in a 14-year-old girl [39]. An excellent clinical response to pan-TRK inhibitors was also observed in two additional patients with ETV6-NTRK3 fusion-positive secretory breast carcinoma (an 8-year-old girl and a 26-year-old man) [24].

In our patient, 4 cycles of EC regimens and 2 cycles of DC regimens neoadjuvant chemotherapy were first used due to the large size of the tumor (12 cm) and late clinical stage (T4N1M0/IIIB). The tumor had a partial response to EC regimens therapy but progressed on DC regimens. To avoid losing the opportunity for surgical treatment, a modified radical mastectomy was performed immediately. A total of 4 cycles of FEC regimens and 8 cycles of intensive chemotherapy were continuously given. Radiotherapy was carried out on the chest wall and drainage areas. The patient was then switched to intensive chemotherapy with oral Capecitabine for 8 cycles. Regrettably, the patient did not receive targeted therapy with a TRK inhibitor due to no such drugs available for clinical use in China at that time. Multiple brain metastases were found 10 months later, and the overall survival of the patient was 19 months.

## Conclusions

In this paper, we report a case of secretory carcinoma of the breast in a 39-year-old female based on imaging, histopathological pattern, immunophenotype and molecular alteration. The tumor was found to be different from this rare subtype of breast cancer, as described typically with indolent progress and favorable prognosis. In this case, the cancer showed chemo-resistance to neoadjuvant chemotherapy and multiple distant metastases in the brain, with a final unfavorable prognosis. To the best of our knowledge, this is the first report of secretory carcinoma of the breast with multiple metastases in the brain and patient died of these metastases. Further research will be needed to better our understanding of the behavior and best therapeutic measures of this rare tumor.

## Data Availability

The datasets used and/or analyzed during the current study are available from the corresponding author on reasonable request.

## Abbreviations

AR: androgen receptor
DC: Docetaxel/Carboplatin
EC: Epirubicin/Cyclophosphamide
ER: estrogen receptor
FEC: Fluorouracil/Epirubicin/cyclophosphamide
GCDFP-15: gross cystic disease fluid protein 15
HER2: human epidermal growth factor receptor 2
FISH: Fluorescence in-situ hybridization
MRI: magnetic resonance imaging
PAS: periodic acid-Schiff
PR: progesterone receptor
SMA: smooth muscle actin
TRK: tropomyosin receptor kinase

## References

[1] McDivitt RW, Stewart FW (1966). Breast carcinoma in children. JAMA.

[2] Eusebi V, Ichihara S, Vincent-Salomon A (2012). Secretory carcinoma. WHO Classification of Tumors of the Breast.

[3] Laé M, Fréneaux P, Sastre-Garau X, Chouchane O, Sigal-Zafrani B, Vincent-Salomon A (2009). Secretory breast carcinomas with ETV6-NTRK3 fusion gene belong to the basal-like carcinoma spectrum. Mod Pathol.

[4] Osako T, Takeuchi K, Horii R, Iwase T, Akiyama F (2013). Secretory carcinoma of the breast and its histopathological mimics: value of markers for differential diagnosis. Histopathology.

[5] Krausz T, Jenkins D, Grontoft O, Pollock DJ, Azzopardi JG (1989). Secretory carcinoma of the Breast in adults: emphasis on late recurrence and metastasis. Histopathology.

[6] AminMB, Edge SB, Greene FL (2017). AJCC Cancer Staging Manual.

[7] Tavassoli FA, Norris HJ (1980). Secretory carcinoma of the breast. Cancer.

[8] Kuwabara H, Yamane M, Okada S (1998). Secretory breast carcinoma in a 66 years old man. J Clin Pathol.

[9] Altundag KJ (2020). Secretory carcinoma of the breast in postmenopausal women. BUON.

[10] Li D, Xiao X, Yang W, Shui R, Tu X, Lu H, Shi D (2012). Secretory breast carcinoma: a clinicopathological and immunophenotypic study of 15 cases with a review of the literature. Mod Pathol.

[11] Shui R, Cheng Y, Bai Q, Yang W (2017). Secretory breast carcinoma with a papillary-predominant pattern: an unusual morphological variant. Histopathology.

[12] Tognon C, Knezevich SR, Huntsman D, Roskelley CD, Melnyk N, Mathers JA (2002). Expression of the ETV6–NTRK3 gene fusion as a primary event in human secretory breast carcinoma. Cancer Cell.

[13] Terada T, Kawata R, Noro K, Higashino M, Nishikawa S, Haginomori SI (2019). Clinical characteristics of acinic cell carcinoma and secretory carcinoma of the parotid gland. Eur Arch Otorhinolaryngol.

[14] Majewska H, Skalova A, Stodulski D, Klimkova A, Steiner P, Stankiewicz C (2015). Mammary analogue secretory carcinoma of salivary glands: a new entity associated with ETV6 gene rearrangement. Virchows Arch.

[15] Ahn CS, Sangüeza OP (2019). Malignant Sweat Gland Tumors.Hematol Oncol. Clin North Am.

[16] Tsutsui K, Takahashi A, Mori T, Namikawa K, Yamazaki N (2020). Secretory carcinoma of the skin arising on the eyelid, distinguished by immunohistochemical markers and fluorescence in situ hybridization. J Dermatol.

[17] Bortz JG, Zhang PJL, Eagle RC, Yong JJ, Milman T (2018). Secretory Carcinoma of the Lacrimal Gland: A Rare Case Report. Ophthalmic Plast Reconstr Surg.

[18] Chu YH, Dias-Santagata D, Farahani AA, Boyraz B, Faquin WC, Nosé V, Sadow PM (2020). Clinicopathologic and molecular characterization of NTRK-rearranged thyroid carcinoma (NRTC). Mod Pathol.

[19] Knezevich SR, McFadden DE, Tao W, Lim JF, Sorensen PH (1998). A novel ETV6-NTRK3 gene fusion in congenital fibrosarcoma. Nat Genet.

[20] De Braekeleer E, Douet-Guilbert N, Morel F, Le Bris MJ, Basinko A, De Braekeleer M (2012). ETV6 fusion genes in hematological malignancies: a review. Leuk Res.

[21] Sasaki E, Masago K, Fujita S, Suzuki H, Hanai N, Hosoda W. Salivary Secretory Carcinoma Harboring a Novel ALK Fusion: Expanding the Molecular Characterization of Carcinomas Beyond the ETV6 Gene. Am J Surg Pathol. 2020 Jul;44(7):962–9. 10.1097/PAS.0000000000001471 32205481

[22] Black M, Liu CZ, Onozato M, Iafrate AJ, Darvishian F, Jour G, Cotzia P. Concurrent Identification of Novel EGFR-SEPT14 Fusion and ETV6-RET Fusion in Secretory Carcinoma of the Salivary Gland. Head Neck Pathol. 2020 Sep;14(3):817–21. 10.1007/s12105-019-01074-6 PMC7413937 31502214

[23] Krings G, Joseph NM, Bean GR, Solomon D, Onodera C, Talevich E (2017). Genomic profiling of breast secretory carcinomas reveals distinct genetics from other breast cancers and similarity to mammary analog secretory carcinomas. Mod Pathol.

[24] Hoda RS, Brogi E, Pareja F, Nanjangud G, Murray MP, Weigelt B (2019). Secretory carcinoma of the breast: clinicopathologic profile of 14 cases emphasising distant metastatic potential. Histopathology.

[25] Krohn M, Trams G, Brandt G (1989). Secretory breast cancer. A special morphologic entity, especially in children and young females. Geburtshilfe Frauenheilkd.

[26] Herz H, Cooke B, Goldstein D (2000). Metastatic secretory breast cancer. Non-responsiveness to chemotherapy: case report and review of the literature. Ann Oncol.

[27] Woto-Gaye G, Kasse AA, Dieye Y, Toure P, Demba Ndiaye P (2004). Secretory breast carcinoma in a man. A case report with rapid evolution unfavorable. Ann Pathol.

[28] Arce C, Cortes-Padilla D, Huntsman DG, Miller MA, Duennas-Gonzalez A, Alvarado A (2005). Secretory carcinoma of the breast containing the ETV6-NTRK3 fusion gene in a male: case report and review of the literature. World J Surg Oncol.

[29] Anderson P, Albarracin CT, Resetkova E (2006). A large, fungating breast mass. Secretory carcinoma with apocrine differentiation. Arch Pathol Lab Med.

[30] Wong M, Jara-Lazaro AR, Hui Ng RC, Tiong Lim AS, Cheok PY, Lim TH (2012). ETV6 disruption does not predict indolent clinical behavior in secretory breast carcinoma. Breast J.

[31] Del Castillo M, Chibon F, Arnould L, Croce S, Ribeiro A, Perot G (2015). Secretory Breast Carcinoma: A Histopathologic and Genomic Spectrum Characterized by a Joint Specific ETV6-NTRK3 Gene Fusion. Am J Surg Pathol.

[32] Lian J, Wang XJ, Xu EW, Wang LX, Bu P, Wang JF (2017). Secretory breast carcinoma with liver metastatic: report of a case. Zhonghua Bing Li Xue Za Zhi.

[33] Tokunaga M, Wakimoto J, Muramoto Y, Sato E, Toyohira O, Tsuchimochi A (1985). Juvenile secretory carcinoma and juvenile papillomatosis. Jpn J Clin Oncol.

[34] Wolberg WH, Street WN, Heisey DM, Mangasarian OL (1995). Computer-derived nuclear “grade” and breast cancer prognosis. Anal Quant Cytol Histol.

[35] Kontzoglou K, Palla V, Karaolanis G, Karaiskos I, Alexiou I, Pateras I (2013). Correlation between Ki67 and breast cancer prognosis. Oncology.

[36] Richard G, Hawk JC, Baker AS, Austin RM (1990). Multicentric adult secretory breast carcinoma: DNA flow cytometric findings, prognostic features, and review of the world literature. J Surg Oncol.

[37] Sharma V, Anuragi G, Singh S, Patel P, Jindal A, Sharma RG. Secretory Carcinoma of the Breast: Report of Two Cases and Review of the Literature. Case Rep Oncol Med. 2015; 2015:581892. 10.1155/2015/581892 PMC4506839 26236516

[38] Cocco E, Scaltriti M, Drilon A (2018). NTRK fusion-positive cancers and TRK inhibitor therapy. Nat Rev Clin Oncol.

[39] Shukla N, Roberts SS, Baki MO, Mushtaq Q, Goss PE, Park BH, et al. Successful Targeted Therapy of Refractory Pediatric ETV6-NTRK3 Fusion-Positive Secretory Breast Carcinoma. JCO Precis Oncol. 2017;2017:PO.17.00034. 10.1200/PO.17.00034 PMC5880290 29623306

